# Taxonomic context and genomic architecture jointly shape expression divergence across animals

**DOI:** 10.1093/evolut/qpag094

**Published:** 2026-05-23

**Authors:** Perry A LaBoone, Antara Anika Piya, Raquel Assis

**Affiliations:** Department of Electrical Engineering and Computer Science, Florida Atlantic University, Boca Raton, Florida, United States; Department of Electrical Engineering and Computer Science, Florida Atlantic University, Boca Raton, Florida, United States; Department of Electrical Engineering and Computer Science, Florida Atlantic University, Boca Raton, Florida, United States; Department of Biomedical Engineering, Florida Atlantic University, Boca Raton, Florida, United States; Institute for Human Health and Disease Intervention, Florida Atlantic University, Boca Raton, Florida, United States

**Keywords:** gene expression, evolution, genome architecture, nested genes, neural network

## Abstract

Gene expression divergence is a major source of phenotypic variation, yet the factors that shift regulatory optima remain incompletely understood. In particular, it is unclear how broad taxonomic differences and local genomic architecture interact to shape the tempo and mode of expression evolution. Here, we analyze single-copy orthologs between species pairs in *Drosophila* and mammals to compare rates and magnitudes of expression divergence, assess the influence of genome architecture, and evaluate tissue-specific and functional patterns. Using a computational framework that predicts expression divergence and species-specific expression optima, we identify markedly higher divergence rates in *Drosophila* than in mammals, consistent with theoretical expectations and prior empirical work. Contrary to expectations, genes located in nested structures, in which one gene lies entirely within the intron of another, were no more likely to diverge than unnested genes in either taxon. However, when divergence occurred in *Drosophila*, nested genes exhibited larger shifts in expression optima, with the strongest effects among internal genes. Divergence rates were also higher among young than old nested genes in both taxa, although magnitudes of expression shifts were indistinguishable, suggesting that nesting contributes to early regulatory instability but does not typically trigger large regulatory changes. Tissue-level and functional analyses further revealed taxon- and architecture-specific signatures of expression divergence, including contrasting patterns across reproductive and neural tissues, as well as enrichment of core regulatory processes among unnested genes and enrichment of drug and xenobiotic metabolism among nested genes in *Drosophila*. Collectively, these findings highlight how taxonomic context and genome architecture can shape expression evolution in distinct and measurable ways, giving rise to contrasting patterns of regulatory divergence.

## Introduction

Understanding how gene expression evolves between species is central to explaining phenotypic diversity and the mechanisms that generate new traits. Comparative transcriptomic studies across taxonomic groups show that expression divergence varies widely among genes and tissues, and that this heterogeneity carries functional and evolutionary significance (Brawand et al., 2011; Cardoso-Moreira et al., 2019; Coolon et al., 2014; Khaitovich et al., 2006; Perry et al., 2012). Although expression levels of orthologous genes often diverge substantially over evolutionary time, the factors that determine which genes maintain conserved expression profiles and which diverge rapidly remain unclear. Several evolutionary mechanisms may account for this variation. Under neutral drift, expression divergence arises through stochastic changes in regulatory elements (Khaitovich et al., 2004; Kimura, 1991; Whitehead & Crawford, 2006). Compensatory changes can also generate divergence when mutations in one regulatory component are offset by others, preserving overall function while altering regulatory interactions (Landry et al., 2005; Wittkopp & Kalay, 2012; Wittkopp et al., 2004). In addition, lineage-specific selection can drive shifts in expression under distinct ecological or physiological pressures (Carroll, 2005; Hirata et al., 2022). Together, these mechanisms provide a framework for explaining why some genes maintain conserved expression profiles, whereas others diverge more rapidly.

One axis along which divergence patterns vary is taxonomic context, which captures lineage-specific differences in genome size and structure, effective population size, and regulatory architecture. For example, *Drosophila* and mammals often vary markedly in these features, all of which can influence the tempo and mode of expression evolution (Adams et al., 2000; Beckenbach et al., 1993; Hahn & Wray, 2002; Jensen & Bachtrog, 2011; Lynch & Conery, 2003; modENCODE Consortium et al., 2010; Moore et al., 2020; Signor & Nuzhdin, 2018). Prior comparative studies suggest that genome-wide expression divergence differs across these clades (Brawand et al., 2011; Cardoso-Moreira et al., 2019; Coolon et al., 2014; Perry et al., 2012), but systematic cross-taxon analyses remain limited, particularly those incorporating tissue-specific patterns (Romero et al., 2012; Wray, 2007). Such contrasts are essential for interpreting lineage-specific signatures of divergence and for contextualizing more specialized genomic features.

A second dimension along which expression divergence varies is genomic architecture. Structural features such as gene order, regulatory neighborhoods, chromatin states, and physical arrangements along chromosomes can influence the accessibility and coordination of regulatory elements (Bell & Spector, 2011; Dixon et al., 2012; Hurst et al., 2002; Kundaje et al., 2015; modENCODE Consortium et al., 2010; Moore et al., 2020; Rowley & Corces, 2018). One of the most striking architectural configurations is the nested gene structure, in which one gene resides entirely within the intron of another (Henikoff et al., 1986; Veeramachaneni et al., 2004; Yu et al., 2005). Nested structures represent the most common form of protein-coding overlap in metazoan genomes, comprising approximately 10% of genes in *Drosophila* and 5% of genes in mammals (Assis et al., 2008). Prior work indicates that nested genes often originate through relocation events that place genes into introns (Assis et al., 2008). Because relocations expose genes to new regulatory environments and, in nested constructs, may introduce transcriptional interference (Assis et al., 2008; Crampton et al., 2006; Da Lage et al., 2003; Osato et al., 2007; Yu et al., 2005), they are thought to influence gene expression. However, a recent study of a small subset of relocated genes in *Drosophila* found that only 23% showed evidence of expression divergence (Piya et al., 2023). This discrepancy raises a broader question: Do relocations generally spur expression divergence, and how do such changes compare with genome-wide background levels?

Adding to this puzzle, previous studies show that nested genes follow strikingly different evolutionary trajectories in *Drosophila* and mammals (Assis, 2016, 2021). In *Drosophila*, nested genes tend to exhibit greater expression divergence than unnested genes, whereas in mammals the two groups are largely comparable. Yet despite these taxon-specific differences, both groups show a consistent pattern in which internal nested genes exhibit greater expression divergence than their external host genes (Assis, 2016, 2021). This pattern suggests that transcriptional interference or regulatory conflict may impose selective pressures favoring either rapid divergence or swift loss of internal genes whose expression overlaps extensively with that of their external hosts. Though these findings highlight nested structures as a potentially important driver of regulatory evolution, their consequences for genome-wide patterns of expression divergence have not been systematically evaluated across taxa.

Here, we investigate how taxonomic context and genomic architecture, particularly nested structures, shape genome-wide patterns of expression divergence. To address these questions, we analyze single-copy orthologs between pairs of species in two deeply diverged clades: *Drosophila* and mammals. We use the neural network PiXi to estimate expression optima and predict expression divergence between orthologous genes (Piya et al., 2023). From these estimates, we compare rates of expression divergence, shifts in expression optima, tissue-specific divergence patterns, and functional enrichment between unnested and nested genes within each species pair. These analyses provide a framework for understanding how taxonomic context and genomic architecture together influence the evolution of gene expression.

## Methods

### Data collection and processing

We analyzed gene expression divergence between *D. melanogaster* and *D. pseudoobscura*, which diverged approximately 54.9 million years ago (Tamura et al., 2004), and between human (*Homo sapiens*) and mouse (*Mus musculus*), which diverged approximately 75 million years ago (Chinwalla et al., 2002). We selected these species pairs because they represent well-characterized model systems with high-quality matched expression datasets and reliable one-to-one ortholog and nested gene annotations. Although they differ in evolutionary timescales and other lineage-specific properties, all analyses of genomic architecture (e.g., comparisons of unnested and nested genes) were conducted within each taxon, such that genes were evaluated across a shared divergence interval. Differences in evolutionary distance between taxa may therefore influence overall rates of expression divergence, but do not affect inference of architectural effects within each taxon.

Tables of quantile-normalized, log-transformed RNA-seq abundances measured in fragments per kilobase of transcript per million mapped fragments (FPKM) from carcass, female head, male head, ovary, testis, and accessory gland tissues in *D. melanogaster* and *D. pseudoobscura* were downloaded from the Dryad repository associated with Assis (2019) at https://datadryad.org/dataset/doi:10.5061/dryad.742564m. Analogous expression tables from brain, heart, lung, colon, liver, kidney, spleen, and testis tissues in human and mouse were downloaded from Expression Atlas (Kapushesky et al., 2010) at https://www.ebi.ac.uk/gxa/home/. These expression values represent normalized measurements derived from biological samples in each species and reflect aggregated expression levels across individuals or replicates in the original studies.

Tables of 1:1 orthologs were sourced from FlyBase (Öztürk Çolak et al., 2024) for *Drosophila* and Ensembl BioMart (Durinck et al., 2005, 2009) for mammals. We focused on single-copy genes to ensure unambiguous orthology and avoid confounding effects of gene duplication. We further restricted analyses to genes expressed in both species of each pair. In total, there were 8,289 1:1 orthologs between *D. melanogaster* and *D. pseudoobscura* and 9,369 between human and mouse.

Nested gene annotations were obtained from Assis (2016) for *Drosophila* and Assis (2021) for mammals. To evaluate the influence of genomic architecture on expression divergence, we initially partitioned these orthologs into two subsets: genes unnested in both species of a taxon, and genes that are nested in either one or both species. This classification captures the overall contrast between unnested and nested architectures; distinctions between genes nested in one versus both species were considered separately in subsequent analyses of nesting age. In total, 1,129 genes were nested in at least one *Drosophila* species, and 513 were nested in at least one mammalian species.

Nested genes were further classified as internal or external: internal nested genes are those whose entire coding sequence lies within an intron of another gene, whereas external nested genes are the host genes that contain one or more internal genes within their introns. In *Drosophila*, there were 659 internal and 470 external nested genes, reflecting the common occurrence of Russian-doll-like multi-level nesting (Assis et al., 2008; Yu et al., 2005). In mammals, there were 247 internal and 266 external nested genes, consistent with the lower overall frequency of nesting and the relative rarity of Russian-doll-like nested architectures in this taxon (Assis et al., 2008).

To assess how the evolutionary age of nesting influences expression divergence, we also subdivided nested genes into young and old classes. Classifications of young and old nested genes were obtained from Assis (2016) for *Drosophila* and Assis (2021) for mammals. Young nested genes were defined as those inferred by parsimony to have acquired their nested arrangement in a single lineage after divergence, such that the gene is nested in one species of the pair but unnested in the other species and in all outgroups considered. Old nested genes were defined as those whose nested arrangement is shared by both species and therefore inferred to predate their divergence. In *Drosophila*, 155 nested gene pairs were classified as young and 358 as old, whereas in mammals 384 nested gene pairs were young and 745 were old.

### Prediction of expression divergence and optima

The PiXi neural network (Piya et al., 2023) was used to predict species-specific expression optima and expression divergence between all orthologs in our study. For training, we generated 40,000 simulated observations, evenly divided between the two PiXi expression divergence classes (“conserved” and “diverged”). Simulations followed the procedure described in Piya et al. (2023), with OU parameters sampled from $\log _{10}(\theta ) \in [0,5]$, $\log _{10}(\alpha ) \in [0,3]$, and $\log _{10}(\sigma ^2) \in [-2,3]$. These ranges span several orders of magnitude in expression optima, selection strength, and evolutionary variance, ensuring that the training data capture a broad range of evolutionary regimes. As in the original implementation (Piya et al., 2023), we trained a two-layer neural network with a batch size of 5,000 for 500 epochs, with 25 values of $\lambda$ uniformly sampled from $\log _{10}(\lambda ) \in [-12,-3]$ and $\gamma \in \lbrace 0, 0.1, \ldots , 1.0\rbrace$. An elastic net penalty was applied to network weights, with $\lambda$ controlling the overall regularization strength and $\gamma$ specifying the mixing ratio between L1 and L2 components.

All 1:1 orthologs and their matched expression data were provided as input to the trained PiXi model, which estimated species-specific expression optima ($\theta$) and assigned each ortholog pair to either the conserved or diverged class (Piya et al., 2023). Pairs were classified as conserved when the two species shared the same predicted expression optimum and as diverged when their species-specific optima differed (Piya et al., 2023).

### Statistical analyses

All statistical analyses were performed in R (R Core Team, 2022) using the Posit Cloud IDE (R Studio Team, 2025). Two-tailed binomial tests were conducted with the binom.test() function (Abdi, 2007) to compare frequencies of all, unnested, and nested genes between *Drosophila* and mammals, as well as frequencies of internal vs. external nested genes and young vs. old nested genes within each taxon. For each test, we set the number of successes *x* to the number of genes in the focal category, the number of trials *n* to the total number of genes, and the null probability of success *p* to 0.5.

Two-tailed permutation tests, implemented with the tidyverse package (R Core Team, 2022; R Studio Team, 2025), were used to evaluate differences between distributions of unnested and nested genes, internal and external nested genes, and young and old nested genes. For each comparison, we calculated the absolute differences in $\theta$ across orthologous pairs and used the mean of these values as the test statistic. Group labels were permuted 10,000 times to generate the null distribution, and empirical *p*-values were defined as the proportion of permuted statistics greater than or equal to the observed value. For tissue-level analyses, *p*-values were Bonferroni-corrected for the number of tissues compared (six in *Drosophila* and eight in mammals) using the p.adjust() function in the stats package (R Core Team, 2022; R Studio Team, 2025).

### Functional enrichment analyses

We used the DAVID Functional Annotation Tool (Huang et al., 2009; Sherman et al., 2022) to evaluate biological functions associated with diverged orthologs in each taxon. We performed two analyses for each taxon: one using diverged unnested genes as the target set and another using diverged nested genes as the target set. For all runs, we used the genome-wide background provided by DAVID. Enrichment was assessed across all annotation clusters available in DAVID. Statistical significance was determined using Fisher’s exact tests with Benjamini-Hochberg FDR correction. This framework allowed us to identify whether diverged orthologs in unnested versus nested contexts were associated with particular biological processes or pathways.

## Results

### Effects of taxonomic context on rates of expression divergence

We first compared the proportions of diverged genes between species pairs. Across all genes, expression divergence was substantially more frequent in *Drosophila* (14.79%) than in mammals (3.97%; $p = 1.97 \times 10^{-253}$, binomial test). A similar contrast was observed for unnested genes, with 15.16% diverged in *Drosophila* and 3.97% in mammals ($p = 1.88 \times 10^{-251}$, binomial test). This pattern aligns with theoretical expectations arising from the larger effective population size of *Drosophila* relative to mammals (Beckenbach et al., 1993; Jensen & Bachtrog, 2011; Lynch & Conery, 2003), as well as with empirical evidence for elevated rates of adaptive protein-coding and regulatory sequence evolution (Britten, 1986; Carroll, 2005; Moriyama, 1987) and rapid, pervasive expression evolution (Coolon et al., 2014; Nourmohammad et al., 2017) in *Drosophila*.

We next examined whether these patterns differed by genomic architecture. Among nested genes, 12.42% were diverged in *Drosophila*, a significantly smaller proportion than the 15.16% observed for unnested genes ($p = 1.07\times 10^{-2}$, binomial test). In contrast, 4.04% of nested mammalian genes were diverged ($p = 6.39 \times 10^{-11}$, binomial test), which was not significantly different from the 3.97% observed for unnested genes ($p = 0.91$, binomial test). Thus, nested genomic architecture was associated with a reduced likelihood of expression divergence in *Drosophila* and showed no detectable effect in mammals.

### Effects of genomic architecture on expression divergence

**Figure 1.**
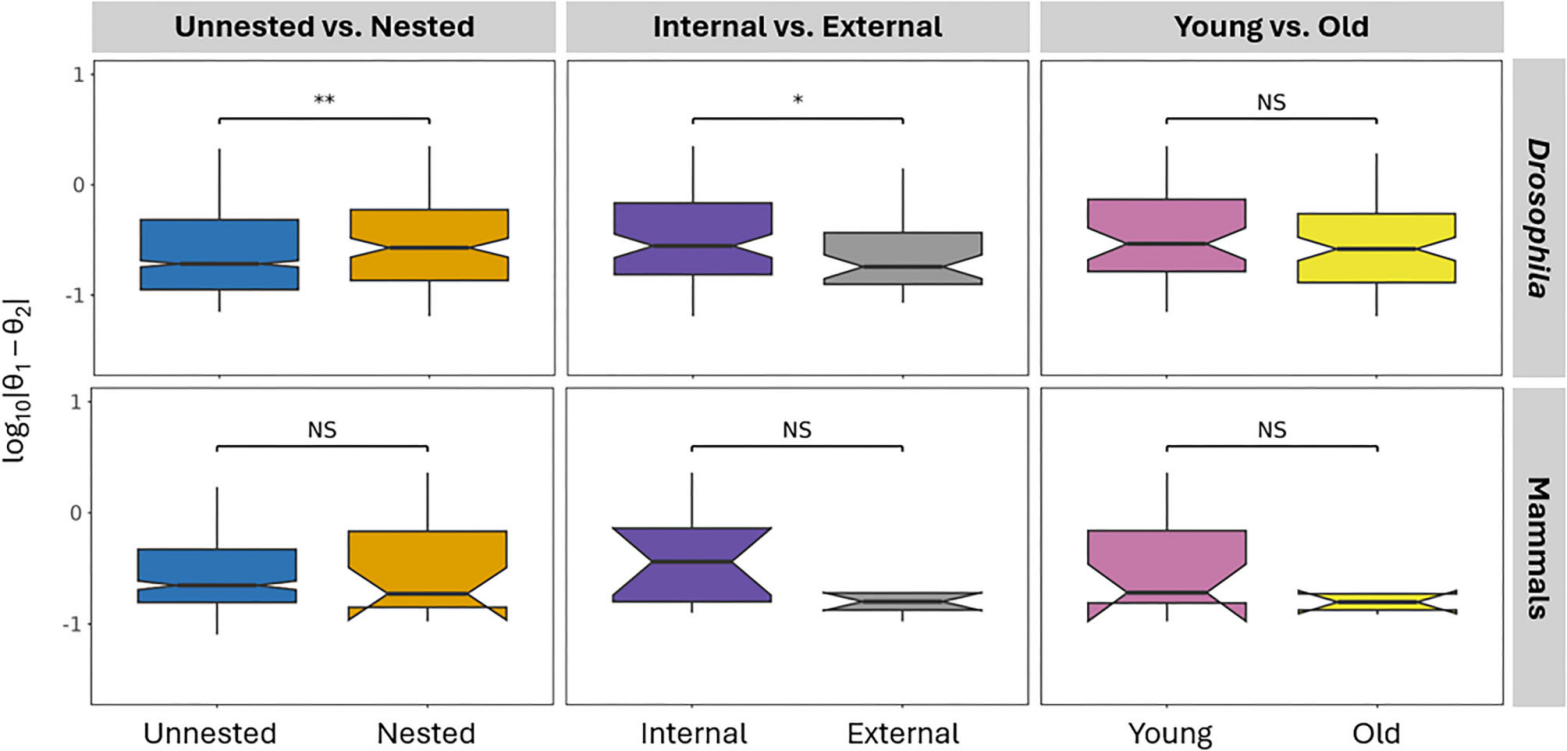
Magnitudes of expression divergence for unnested and nested orthologs. Box plots show distributions of absolute differences between species-specific expression optima for unnested and nested genes (left), internal and external nested genes (middle), and young and old nested genes (right) in *Drosophila* (top) and mammals (bottom). *$p< 0.05$, **$p< 0.01$, ***$p< 0.001$, NS = not significant (see *Methods*).

Our finding that gene nesting does not influence the rate of expression divergence in mammals is consistent with previous work showing that mammalian genes typically retain their expression profiles after nesting (Assis, 2021). However, in *Drosophila*, the reduced rate of expression divergence among nested genes initially appeared to conflict with an earlier report of elevated expression divergence following nesting in this taxon (Assis, 2016). To investigate this apparent discrepancy, we compared differences in expression optima between diverged unnested and nested genes in each species pair (Figure 1). Consistent with prior reports (Assis, 2016, 2021), nested genes exhibited larger shifts in expression optima than unnested genes in *Drosophila* ($p = 8.3 \times 10^{-3}$, permutation test), whereas no significant difference was observed in mammals ($p = 0.92$, permutation test; Figure 1, left).

We then asked whether internal and external nested genes contributed differently to these patterns. Our analysis revealed that expression divergence was significantly more frequent among internal (14.15%) than external (9.98%) genes in *Drosophila* ($p = 8.07 \times 10^{-4}$, binomial test), whereas in mammals, internal and external genes exhibited similar frequencies of divergence (4.44% vs. 3.60%; $p = 0.41$, binomial test). Internal genes also tended to display greater shifts in expression optima in *Drosophila* ($p = 0.03$, permutation test), but not in mammals ($p = 0.11$, permutation test; Figure 1, middle). These results indicate that although nested genes may not diverge more frequently than unnested genes in either taxon, the divergence that does occur in *Drosophila* is often pronounced and biased toward internal genes, consistent with prior work (Assis, 2016).

To examine whether the evolutionary age of nested structures influenced these outcomes, we compared rates of expression and shifts in expression optima between young and old nested genes. In *Drosophila*, divergence rates were higher in young (14.48%) than in old (11.41%) nested genes, but this difference was not statistically significant ($p = 0.07$, binomial test). However, in mammals, divergence was significantly more frequent in young (5.21%) than in old (2.35%) nested genes ($p = 3.49 \times 10^{-3}$, binomial test). Despite these differences in rates, young and old nested genes did not differ in the magnitudes of their shifts in expression optima in either taxon ($p = 0.28$ in *Drosophila*, $p = 0.39$ in mammals, permutation tests; Figure 1, right). Together, these findings suggest that expression divergence is not generally driven by nesting itself, but rather reflects the selective retention of nesting events involving genes that were already sufficiently distinct.

### Functional characterization of expression divergence

**Figure 2.**
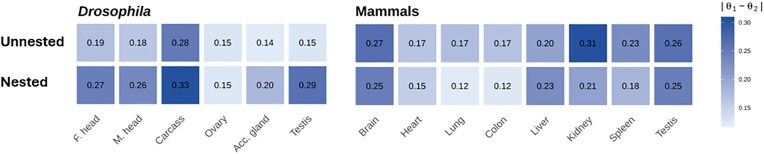
Magnitudes of tissue-specific expression divergence for unnested and nested orthologs in *Drosophila* and mammals. Heatmaps depict absolute differences between species-specific expression optima across individual tissues for unnested (top) and nested (bottom) genes in *Drosophila* (left) and mammals (right).

To place expression divergence in a functional context, we examined tissue-specific shifts in expression optima among diverged orthologs within each species pair (Figure 2). Among *Drosophila* unnested genes, expression divergence was highest in carcass and consistently lowest across the three sex tissues (ovary, testis, and accessory gland). In mammals, divergence of unnested genes was highest in kidney and lowest in heart, lung, and colon. Tissue-specific patterns also differed between unnested and nested genes. In *Drosophila*, nested genes again showed the highest divergence in carcass but were followed by markedly elevated divergence in testis, approximately twofold higher than that observed for unnested genes; female and male head and accessory gland also exhibited nearly 1.5-fold higher divergence in nested relative to unnested genes. In mammals, nested genes displayed the highest divergence in brain and testis and the lowest divergence in lung and colon, consistent with prior studies (Brawand et al., 2011; Cardoso-Moreira et al., 2019; Khaitovich et al., 2004). Together, these results demonstrate that tissue-specific expression divergence varies across both taxa and genomic contexts.

The observed tissue-specific differences prompted us to examine whether expression divergence is associated with particular biological functions. To address this question, we performed functional enrichment analyses of diverged genes within each species pair. To capture a broad range of functional categories, we incorporated annotations from the Gene Ontology (GO) Consortium (The Gene Ontology Consortium, 2021), curated pathways from the Kyoto Encyclopedia of Genes and Genomes (KEGG) (Kanehisa et al., 2016), signaling and regulatory networks from BioCarta (BioCarta, 2025), and protein domain features from InterPro (Blum et al., 2025) and the Simple Modular Architecture Research Tool (SMART) (Letunic & Bork, 2018). Enrichment was assessed by testing whether each target set of diverged genes was overrepresented in specific functional categories relative to the genome-wide background (see *Methods*).

**Figure 3.**
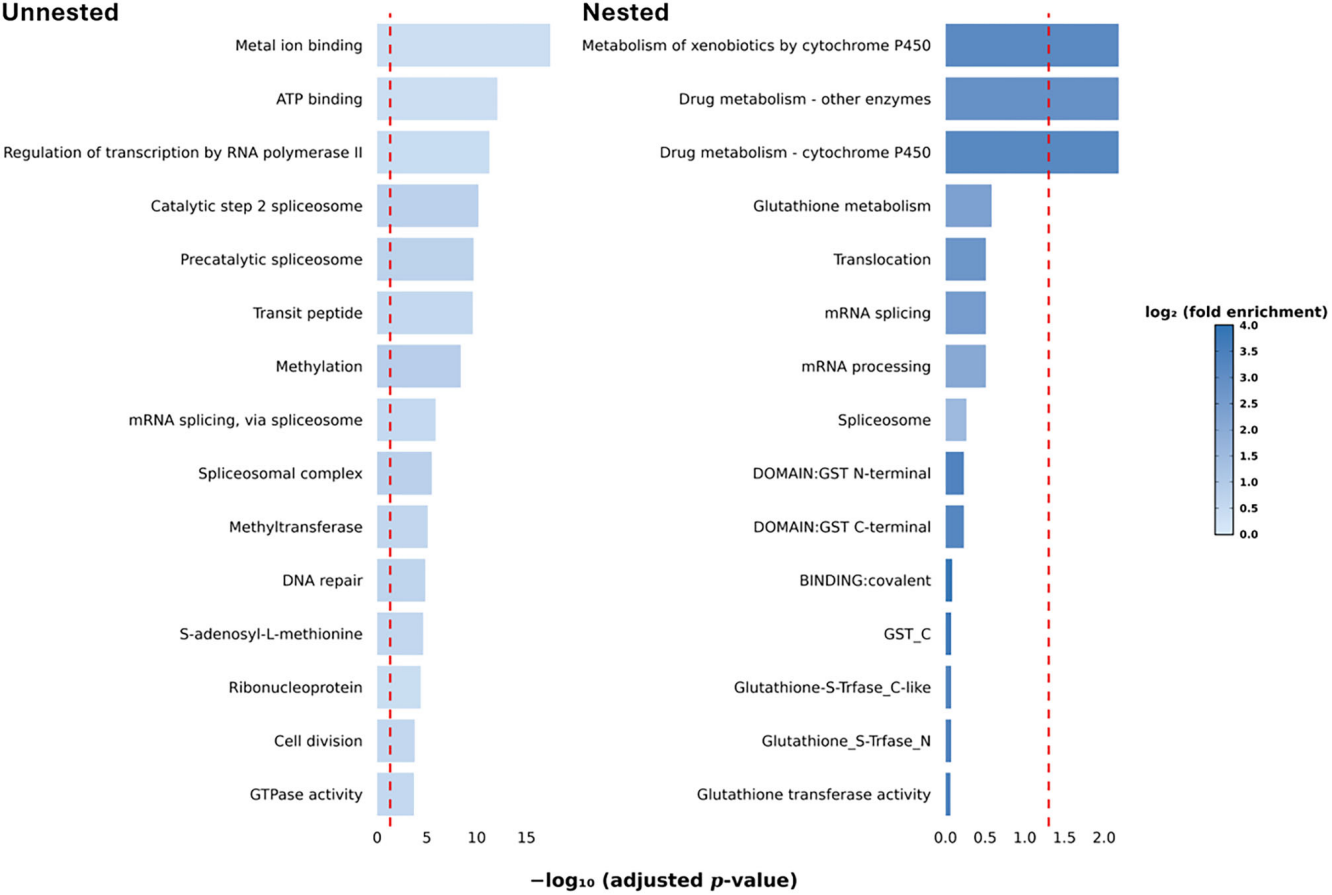
Visualization of the top 15 enriched terms for diverged *Drosophila* genes. Bar plots show terms ranked by Benjamini-adjusted *p*-values for unnested (left) and nested (right) genes, with the red dashed line indicating the threshold for statistical significance (see *Methods*). The complete sets of enriched terms are provided in Tables S1 and S3.

Our functional enrichment analyses revealed distinct taxon- and architecture-dependent patterns. Among *Drosophila* diverged unnested genes, we observed significant enrichment for transcriptional regulation and RNA processing (Table S1), whereas mammalian diverged unnested genes were enriched primarily for cellular component terms related to mitochondrial and membrane localization (Table S2). In contrast, diverged nested genes in *Drosophila* exhibited enrichment for drug and xenobiotic metabolism, particularly cytochrome P450-mediated processes (Coelho et al., 2015) (Table S3), while no statistically significant functional enrichment was detected among mammalian diverged nested genes. Visualization of the top 15 enriched terms clarifies the architectural contrast within *Drosophila*, illustrating that nested genes exhibit fewer but substantially larger enrichment signals than unnested genes (Figure 3).

To investigate functional themes associated with strong expression divergence, we examined the top-ranked unnested and nested ortholog pairs in each taxon, defined by the largest absolute differences in estimated expression optima. Among *Drosophila* unnested genes, the most divergent exemplar was Oligosaccharyltransferase Complex Subunit 4 (*Ost4*, FBgn0053774), a small ER transmembrane protein essential for N-linked glycosylation and protein maturation (Dumax-Vorzet et al., 2013; Katoh & Tiemeyer, 2013; Ruiz-Canada et al., 2009). The top mammalian unnested gene was Alpha-2-Macroglobulin (*A2M*, ENSG00000175899), a broad-spectrum protease inhibitor involved in cytokine transport, immune regulation, and proteostasis, with established links to inflammatory and neurodegenerative processes (Du et al., 1997; French et al., 2008; Wong & Dessen, 2014). Among *Drosophila* nested genes, the most divergent gene was Ubiquinol-Cytochrome c Reductase 11 kDa Subunit-Like (*UQCR-11L*, FBgn0050354), a mitochondrial complex III subunit previously identified as highly divergent in an analysis of a smaller set of relocated genes (Piya et al., 2023). In mammals, the top nested exemplar was Ribosomal Protein S27 (*RPS27*, ENSG00000177954), a zinc-binding component of the 40S ribosomal subunit involved in translation, rRNA processing, and stress response signaling that is frequently dysregulated in human disease (Gazda et al., 2006; Xiong et al., 2014; Zhang et al., 2003). Together, these cases highlight the distinct molecular functions associated with pronounced expression divergence and illustrate how genomic architecture can shape gene-specific evolutionary trajectories.

## Discussion

Our results suggest that broad taxonomic context and local genomic architecture leave distinct, interacting signatures on the evolution of gene expression in the species pairs analyzed here. Expression divergence was nearly four times more frequent in *Drosophila* than in mammals, consistent with expectations from population-genetic theory (Beckenbach et al., 1993; Jensen & Bachtrog, 2011; Lynch & Conery, 2003) and extensive evidence for rapid regulatory evolution in *Drosophila* (Britten, 1986; Carroll, 2005; Coolon et al., 2015; Moriyama, 1987; Nourmohammad et al., 2017). At the same time, genomic architecture added another layer of heterogeneity. In *Drosophila*, nested genes were less likely than unnested genes to diverge, yet those that did diverge displayed larger shifts in expression optima, with internal genes showing the strongest effects. In contrast, mammalian nested and unnested genes showed similar frequencies and magnitudes of expression divergence.

In both taxa, young nested genes diverged more frequently than old nested genes, suggesting a transient period of regulatory instability after nesting. However, the size of expression shifts was similar between young and old nested genes, indicating that nesting does not typically initiate large expression changes but instead shapes expression trajectories once divergence begins. Several hypotheses could explain this pattern. Under the transcriptional interference hypothesis (Crampton et al., 2006; Da Lage et al., 2003; Osato et al., 2007), newly nested genes may experience regulatory conflict that increases the likelihood of early divergence, with older pairs stabilizing once interference is reduced, consistent with observations in *Drosophila* and mammals (Assis, 2016, 2021). In contrast, co-regulation models (Davies et al., 2004; Furia et al., 1993; Habib et al., 1998; Henikoff & Eghtedarzadeh, 1987; Jaworski et al., 2007) predict that only compatible nested pairs persist, which could also produce lower divergence frequencies in older genes without affecting divergence magnitude. Finally, the neutral insertion hypothesis (Habib et al., 1998; Lynch, 2002, 2006, 2007a,b; Lynch & Conery, 2003; Yi, 2006) is consistent with the similar magnitudes of divergence across age classes but does not readily explain the strong age-dependent difference in divergence frequency that we observe. Tissue-level analyses further revealed that divergence is distributed unevenly across organ systems in a taxon- and architecture-specific manner, and functional enrichment patterns mirrored these differences. Together, these findings illustrate that genome-wide patterns of expression divergence emerge from the interplay between lineage-specific biological constraints and the fine-scale physical organization of genomes.

The contrasting divergence patterns between *Drosophila* and mammalian species pairs may reflect long-standing differences in effective population size, regulatory complexity, and the strength of purifying selection (Beckenbach et al., 1993; Jensen & Bachtrog, 2011; Lynch & Conery, 2003). In addition, differences in divergence time and generation time between these taxa may contribute to the magnitude of differences we observe, as *Drosophila* lineages are separated by a substantially larger number of generations than mammalian lineages. Regulatory evolution in *Drosophila* has been shown to proceed rapidly due to efficient selection and turnover of *cis*-regulatory elements (McGregor et al., 2007; Signor & Nuzhdin, 2018; Wittkopp & Kalay, 2012), and the greater divergence in *Drosophila* may reflect both faster regulatory turnover and the larger number of generations that have accumulated since lineage separation. By contrast, mammalian expression profiles are generally more constrained (Brawand et al., 2011; Khaitovich et al., 2006; Perry et al., 2012), consistent with both slower regulatory evolution and fewer generational opportunities for divergence to accumulate.

Within each taxon, genomic architecture further structured divergence. The larger expression shifts observed among *Drosophila* nested genes—particularly internal genes—are consistent with hypotheses that transcriptional interference, competition for regulatory elements, or altered chromatin states may affect expression dynamics of genes embedded within introns (Assis et al., 2008; Crampton et al., 2006; Da Lage et al., 2003; Osato et al., 2007). Elevated divergence rates among young nested genes in both taxa likewise parallel findings that relocated or recontextualized genes often experience rapid expression changes before stabilizing (Assis, 2016; Assis & Bachtrog, 2013; Bhutkar et al., 2007; Egecioglu & Brickner, 2011; Kaessmann et al., 2009; Meisel, 2009). Because divergence magnitudes did not differ by age, these results imply that architectural context influences how expression evolves after divergence begins rather than determining the extent of that divergence.

A key distinction between taxa emerges when considering internal nested genes. In *Drosophila*, the reduced overall prevalence of divergence among nested genes is consistent with the possibility that many internal genes arrive with expression profiles already sufficiently distinct from those of their external hosts, reducing the likelihood that further divergence is necessary. Conversely, when an internal gene does not arrive with an adequately differentiated regulatory profile, it may undergo rapid and pronounced divergence, producing the large shifts we observe. These patterns suggest, but do not definitively establish, that internal genes in *Drosophila* may follow one of two trajectories: arriving already distinct or rapidly diverging to become so. In mammals, internal genes exhibited neither elevated divergence frequencies nor increased divergence magnitudes relative to nested genes. These results suggest that mammalian internal genes may arrive with their regulatory distinctiveness already established, as they do not exhibit the rapid post-formation divergence trajectory observed in *Drosophila*. This contrast highlights fundamental differences in the evolutionary opportunities and constraints associated with genomic architectural changes across taxa.

Tissue-level analyses showed that expression divergence is distributed unevenly across organ systems in ways that differ between taxa and genomic contexts. These differences may reflect variation in tissue-specific regulatory constraints and functional demands. Among unnested genes, all three *Drosophila* reproductive tissues (testis, accessory gland, and ovary) showed consistently low divergence, whereas in mammals, testis was among the tissues showing the highest divergence. Relative to unnested genes, *Drosophila* nested genes exhibited approximately twofold higher divergence in testis and nearly 1.5-fold higher divergence in female and male head and accessory gland, suggesting that nested gene divergence within this taxon may be associated with reproductive and neural functions. Similarly, mammalian nested genes also displayed their highest divergence in brain and testis, in contrast to the elevated kidney divergence observed for unnested genes. Functional enrichment patterns further supported this view. Unnested genes were enriched for transcriptional regulation and RNA processing in *Drosophila* and for mitochondrial and membrane targeting in mammals, suggesting that different core cellular pathways contribute disproportionately to expression divergence in each taxon. *Drosophila* nested genes were enriched for detoxification and xenobiotic pathways, consistent with the rapid and lineage-specific evolution of environmental response genes (Darragh et al., 2021; Dermauw et al., 2020; Good et al., 2014; Lu et al., 2021; Thomas, 2007). In contrast, mammalian nested genes showed no clear functional enrichment, though top-ranked terms were associated with membrane signaling functions. These tissue-specific and functional differences underscore that taxonomic and architectural contexts together shape the regulatory pressures acting on individual genes.

Our findings motivate several directions for further investigation. Because our analyses are based on a single pairwise comparison in each taxon, the extent to which the observed patterns generalize to other species within and across taxa remains uncertain. Expanding analyses to additional species, particularly those differing in genome size, life history, and effective population size, will be essential for determining whether the architectural patterns observed here are lineage-specific or broadly conserved. More broadly, because our analyses rely on species-level expression profiles, we cannot directly assess within-species variability. Incorporating individual-level expression data would help identify genes that are inherently more variable and therefore more likely to diverge across species. Integrating regulatory sequence evolution, chromatin accessibility, and three-dimensional genome organization could further elucidate mechanisms underlying the amplified expression divergence of internal nested genes, especially in light of emerging links between intragenic architecture and chromatin topology (Bonev & Cavalli, 2016; Rowley & Corces, 2018). Finer-grained tissue resolution, especially in mammals, would improve power to detect subtle architectural effects and reveal whether particular cell types are especially prone to divergence in nested contexts. Connecting expression divergence with phenotypic, ecological, or environmental data may illuminate cases in which architectural constraints or opportunities contribute to adaptive regulatory evolution. Together, these efforts will help clarify the generality, underlying mechanisms, and biological relevance of the patterns reported here.

## Supplementary Material

qpag094_Supplemental_File

## Data Availability

All R code and processed datasets are available at https://doi.org/10.5281/zenodo.20084890.
