# Supplementary material for: Taxonomic context and genomic architecture jointly shape expression divergence across animals
Source: Evolution. 2026 May 23;80(7):1561–70. doi: 10.1093/evolut/qpag094 (PMC13343718; doi:10.1093/evolut/qpag094)
Supplement: qpag094_Supplemental_File [file qpag094_supplemental_file.pdf]

Table S1: Functional enrichment results for “diverged” unnested genes in *Drosophila*.

Source	Term	Adjusted p-value	Fold Enrichment
GOTERM_MF_DIRECT	Metal ion binding	4.13E-18	1.30
GOTERM_MF_DIRECT	ATP binding	8.37E-13	1.30
GOTERM_BP_DIRECT	Regulation of transcription by RNA polymerase II	5.47E-12	1.33
GOTERM_CC_DIRECT	Catalytic step 2 spliceosome	6.58E-11	1.69
GOTERM_CC_DIRECT	Precatalytic spliceosome	2.16E-10	1.64
UP_KW_DOMAIN	Transit peptide	2.45E-10	1.46
GOTERM_BP_DIRECT	Methylation	4.14E-09	1.83
GOTERM_BP_DIRECT	mRNA splicing, via spliceosome	1.34E-06	1.43
GOTERM_CC_DIRECT	Spliceosomal complex	3.34E-06	1.74
UP_KW_MOLECULAR_FUNCTION	Methyltransferase	8.38E-06	1.52
GOTERM_BP_DIRECT	DNA repair	1.49E-05	1.59
UP_KW_LIGAND	S-adenosyl-L-methionine	2.39E-05	1.49
UP_KW_MOLECULAR_FUNCTION	Ribonucleoprotein	4.29E-05	1.31
GOTERM_BP_DIRECT	Cell division	1.71E-04	1.48
GOTERM_MF_DIRECT	GTPase activity	2.13E-04	1.45
UP_KW_MOLECULAR_FUNCTION	Helicase	2.27E-04	1.42
UP_KW_MOLECULAR_FUNCTION	Kinase	2.27E-04	1.22
UP_KW_MOLECULAR_FUNCTION	Helicase	2.27E-04	1.42
GOTERM_MF_DIRECT	RNA polymerase II cis-regulatory region sequence-specific DNA binding	5.63E-04	1.26
UP_KW_CELLULAR_COMPONENT	Mitochondrion	7.62E-04	1.16
GOTERM_MF_DIRECT	Protein serine/threonine kinase activity	1.00E-03	1.40
GOTERM_MF_DIRECT	Protein serine/threonine kinase activity	1.00E-03	1.40
UP_SEQ_FEATURE	DOMAIN:C2H2-type	1.38E-03	1.28
GOTERM_BP_DIRECT	Mitochondrial respiratory chain complex I assembly	1.59E-03	1.74
SMART	ZnF_C2H2	1.82E-03	1.25
SMART	SEC14	1.82E-03	1.80
GOTERM_BP_DIRECT	Protein ubiquitination	3.23E-03	1.36
GOTERM_BP_DIRECT	Circadian rhythm	3.23E-03	1.59
GOTERM_BP_DIRECT	Transcription by RNA polymerase II	3.28E-03	1.72
UP_SEQ_FEATURE	TRANSIT:Mitochondrion	3.43E-03	1.35
UP_KW_DOMAIN	Kelch repeat	3.59E-03	1.99
GOTERM_MF_DIRECT	DNA-binding transcription factor activity, RNA polymerase II-specific	3.88E-03	1.22
GOTERM_MF_DIRECT	mRNA binding	3.88E-03	1.31
UP_KW_DOMAIN	WD repeat	4.03E-03	1.33
GOTERM_BP_DIRECT	DNA-templated DNA replication	4.23E-03	1.95
GOTERM_BP_DIRECT	Endosome transport via multivesicular body sorting pathway	4.49E-03	2.12
GOTERM_MF_DIRECT	Rho-dependent protein serine/threonine kinase activity	4.87E-03	1.57
GOTERM_MF_DIRECT	AMP-activated protein kinase activity	4.87E-03	1.57
GOTERM_MF_DIRECT	Eukaryotic translation initiation factor 2alpha kinase activity	4.87E-03	1.57
GOTERM_MF_DIRECT	Histone H3S28 kinase activity	4.87E-03	1.56
GOTERM_MF_DIRECT	Histone H4S1 kinase activity	4.87E-03	1.56
GOTERM_MF_DIRECT	Histone H2AS1 kinase activity	4.87E-03	1.56
GOTERM_MF_DIRECT	Histone H2BS14 kinase activity	4.87E-03	1.56
GOTERM_MF_DIRECT	Histone H3S57 kinase activity	4.87E-03	1.56
GOTERM_MF_DIRECT	Histone H3T45 kinase activity	4.87E-03	1.56
GOTERM_MF_DIRECT	Histone H2AXS139 kinase activity	4.87E-03	1.56
GOTERM_MF_DIRECT	Histone H3T3 kinase activity	4.87E-03	1.56
GOTERM_MF_DIRECT	Histone H2AS121 kinase activity	4.87E-03	1.56
GOTERM_MF_DIRECT	3-phosphoinositide-dependent protein kinase activity	4.87E-03	1.56
GOTERM_MF_DIRECT	DNA-dependent protein kinase activity	4.87E-03	1.56
GOTERM_MF_DIRECT	Histone H3S10 kinase activity	4.87E-03	1.56
GOTERM_MF_DIRECT	Histone H2BS36 kinase activity	4.87E-03	1.56
GOTERM_MF_DIRECT	Ribosomal protein S6 kinase activity	4.87E-03	1.56
GOTERM_MF_DIRECT	Histone H3T11 kinase activity	4.87E-03	1.56
GOTERM_MF_DIRECT	Histone H3T6 kinase activity	4.87E-03	1.56
GOTERM_MF_DIRECT	Histone H2AT120 kinase activity	4.87E-03	1.56
GOTERM_BP_DIRECT	Mitochondrial translation	4.98E-03	1.43
INTERPRO	SAM-dependent_MTases_sf	5.73E-03	1.48

Source	Term	Adjusted p-value	Fold Enrichment
INTERPRO	Znf_C2H2_sf	5.73E-03	1.26
GOTERM_MF_DIRECT	GTP binding	6.02E-03	1.31
GOTERM_MF_DIRECT	Phosphatidylinositol bisphosphate binding	7.55E-03	1.92
GOTERM_MF_DIRECT	RNA helicase activity	7.55E-03	1.61
UP_KW_MOLECULAR_FUNCTION	Ribosomal protein	8.31E-03	1.27
UP_KW_DOMAIN	Leucine-rich repeat	8.36E-03	1.31
GOTERM_MF_DIRECT	Protein kinase activity	1.01E-02	1.36
UP_KW_DOMAIN	Transmembrane	1.14E-02	1.04
UP_KW_MOLECULAR_FUNCTION	Thiol protease	1.21E-02	1.48
UP_SEQ_FEATURE	DOMAIN:CRAL-TRIO	1.27E-02	1.73
UP_KW_CELLULAR_COMPONENT	Spliceosome	1.32E-02	1.42
GOTERM_MF_DIRECT	Ubiquitin-like ligase-substrate adaptor activity	1.63E-02	1.81
GOTERM_MF_DIRECT	SNARE binding	1.71E-02	1.68
GOTERM_MF_DIRECT	Protein serine kinase activity	1.73E-02	1.40
GOTERM_MF_DIRECT	Protein serine kinase activity	1.73E-02	1.40
GOTERM_BP_DIRECT	Transcription initiation at RNA polymerase II promoter	2.69E-02	1.61
GOTERM_MF_DIRECT	Four-way junction helicase activity	2.81E-02	1.72
GOTERM_CC_DIRECT	Vesicle	2.84E-02	1.50
UP_KW_MOLECULAR_FUNCTION	Serine/threonine-protein kinase	3.01E-02	1.22
UP_KW_MOLECULAR_FUNCTION	Serine/threonine-protein kinase	3.01E-02	1.22
GOTERM_CC_DIRECT	Cul3-RING ubiquitin ligase complex	3.19E-02	2.00
UP_KW_DOMAIN	ANK repeat	3.46E-02	1.38
UP_KW_BIOLOGICAL_PROCESS	Cell cycle	3.50E-02	1.17
GOTERM_MF_DIRECT	Transcription cis-regulatory region binding	3.74E-02	1.37
GOTERM_MF_DIRECT	DNA helicase activity	3.75E-02	1.88
GOTERM_MF_DIRECT	DNA clamp loader activity	3.75E-02	1.47
GOTERM_MF_DIRECT	Cysteine-type deubiquitinase activity	3.91E-02	1.65
GOTERM_MF_DIRECT	Single-stranded 3'-5' DNA helicase activity	4.05E-02	1.70
GOTERM_MF_DIRECT	Double-stranded DNA helicase activity	4.05E-02	1.70
INTERPRO	CRAL-TRIO_dom_sf	4.28E-02	1.67
GOTERM_MF_DIRECT	Pyridoxal phosphate binding	4.62E-02	1.66

Table S2: Functional enrichment results for "diverged" unnested genes in mammals.

Source	Term	Adjusted p-value	Fold Enrichment
GOTERM_CC_DIRECT	Mitochondrion	3.54E-5	2.05
UP_KW_CELLULAR_COMPONENT	Mitochondrion	3.19E-4	1.96
UP_KW_DOMAIN	Transit peptide	3.31E-3	2.49
GOTERM_CC_DIRECT	Membrane	3.64E-3	1.36
GOTERM_MF_DIRECT	S100 protein binding	1.24E-2	19.76
UP_KW_BIOLOGICAL_PROCESS	Ubl conjugation pathway	2.47E-2	1.91
GOTERM_MF_DIRECT	ATP binding	4.97E-2	1.68

Table S3: Functional enrichment results for "diverged" nested genes in *Drosophila*.

Source	Term	Adjusted p-value	Fold Enrichment
KEGG_PATHWAY	Drug metabolism - other enzymes	6.57E-3	7.55
KEGG_PATHWAY	Drug metabolism - cytochrome P450	6.57E-3	9.14
KEGG_PATHWAY	Metabolism of xenobiotics by cytochrome P450	6.57E-3	8.88
